# Association Analysis of MMP-13 (rs2252070) Gene Polymorphism and the Susceptibility to Chronic Periodontitis

**DOI:** 10.7759/cureus.57426

**Published:** 2024-04-01

**Authors:** Shubhangini Chatterjee, Arvina Rajasekar

**Affiliations:** 1 Department of Periodontics, Saveetha Dental College and Hospitals, Saveetha Institute of Medical and Technical Sciences, Saveetha University, Chennai, IND

**Keywords:** dna amplification, genetics, gene polymorphism, mmp-13, chronic generalized periodontitis

## Abstract

Background: Chronic periodontitis is a multifactorial inflammatory condition influenced by genetic factors. Matrix metalloproteinase (MMP)-13, serving as a crucial enzyme involved in extracellular matrix remodeling, is associated with the degradation of periodontal tissues. Therefore, this study assesses the genetic link between the MMP-13 (rs2252070) genetic variation and chronic periodontitis in a Southern Indian demographic.

Methodology: The study was conducted at Saveetha Dental College in Chennai, India. It involved a total of 100 subjects, 50 individuals affected with periodontitis (classified as stage II and above, American Association of Periodontology 2018 criteria) and 50 individuals who were periodontally healthy or were diagnosed as having mild gingivitis. We isolated DNA from the blood samples obtained from the participants. Specific primers that flank the BsrI region of the MMP-13 receptor gene were used in the process of DNA amplification. Subsequently, a restriction fragment length analysis using the BsrI enzyme was carried out for genotyping of the amplicon. Based on the restriction fragment length polymorphism pattern, we obtained certain genotypes. These were further recorded and followed by statistical analysis. We conducted a chi-square test to draw a comparison in terms of their genotype and allele frequencies. We calculated the odds ratio, along with 95% confidence intervals.

Results: The frequency of genotypes and distribution of MMP-13 polymorphism did not exhibit a statistically significant difference at χ2 degrees of freedom (P = 0.913). We inferred from our study that there was no significant difference between the groups concerning homozygous and heterozygous mutant genotypes (AA vs. AG + GG), with a P-value of 0.6871. The observed frequencies of GG (47% vs. 43%) and AG+AA (41% vs. 42%) genotypes did not indicate a significant difference between the groups. Similarly, there was no noteworthy distinction between the A allele (62% vs. 65%) and G allele (38% vs. 35%) in the case and control groups.

Conclusion: The findings of the study reveal that there is no correlation between MMP-13 (rs2252070) gene polymorphism and periodontitis.

## Introduction

Multiple factors contribute to the development of the chronic inflammatory disease known as periodontitis, which leads to the loss of teeth by causing the destruction of the supporting tissues [1]. With a prevalence of 15-20%, it is typically regarded as one of the most prevalent diseases worldwide [2]. The fact that periodontitis has been linked to other severe illnesses, like chronic obstructive pulmonary disease, head and neck cancer, and coronary heart disease, is more concerning [3,4]. To effectively treat periodontitis, early detection and intervention are crucial. At the professional level, it is simple to distinguish between people with destructive periodontitis and healthy people because the primary factors are certain criteria used for clinical diagnosis, including measurements, such as probing depth of pocket, attachment level, bleeding upon probing, plaque index, and evaluation through the analysis of radiographs [5]. Dentists continue to face challenges in treating the initial stages of initiation and/or progression based on the clinical diagnostic criteria [6].

The individual's genetic background plays a key role in determining susceptibility to various diseases and conditions, including periodontitis. There is a strong interaction between the genetic makeup of the host and environmental factors in causing periodontal disease. There are many modifiable risk factors for periodontal disease, such as smoking and diabetes mellitus, whereas genetics is a risk factor that cannot be changed and greatly affects a person's susceptibility to periodontal disease [7]. In diseases like chronic periodontitis, disease susceptibility is influenced by numerous single-nucleotide polymorphisms [8,9]. Matrix metalloprotease (MMP) enzymes are thought to be proteinases derived from the host. They have the potential to be used as gingival crevicular fluid (GCF) biomarkers. MMPs are zymogens that require zinc to function. They degrade and inactivate chemokines and promote cell proliferation, angiogenesis, and apoptosis [10].

Sustaining periodontal health necessitates a delicate balance among enzymes causing the destruction of tissue, such as MMPs and MMP inhibitors. The proteolytic enzymes within the MMP family contribute to the initial remodeling of the matrix and basement membrane, and their participation persists as various diseases advance [11]. The breakdown of the extracellular matrix is orchestrated by a sequence of events that encompass proteinases from both the host and microbial sources. MMPs form a category of proteinases that contribute significantly to this destructive process [12]. Within these proteinases, MMP-13 (collagenase-3) expression is acknowledged as having a role in bone resorption and cartilage destruction in conditions, such as rheumatoid arthritis and osteoarthritis. There is evidence indicating that MMP-13 may also cause alveolar bone destruction in periodontitis [13]. Genetic polymorphisms of MMP genes are pivotal factors in predisposing individuals to different diseases, influencing changes in structure and function [14].

Nevertheless, the outcomes consistently demonstrate inconsistency, and individual studies with limited sample sizes lack the statistical power to thoroughly investigate the genuine association between these genetic polymorphisms and chronic periodontitis [15]. In the present research, we examined the impact of MMP-13 (rs2252070) gene polymorphism on the predisposition to chronic periodontitis and explored the potential correlation between these genetic variations and chronic periodontitis in a southern Indian demographic.

## Materials and methods

This study was conducted at Saveetha Dental College in Chennai, India. One hundred patients seeking periodontal therapy at our periodontics department were randomly chosen for the current investigation. We allocated 50 individuals to the control group (Group A), and the remaining 50 subjects (Group B) were patients diagnosed with periodontitis based on the American Academy of Periodontology (AAP) 2018 criteria.

Inclusion criteria

Group A (control group) were periodontally and systemically healthy individuals. Group B individuals were diagnosed with periodontitis (based on the AAP 2018 criteria) but were systemically healthy.

Exclusion criteria

Immunocompromised individuals, smokers, pregnant females, and patients who underwent periodontal therapy in the last six months were excluded. The Institutional Review Board of Saveetha Dental College gave ethical clearance for carrying out the study (IHEC/SDC/PERIO-2105/23/027). Written informed consent was obtained from patients.

Sample collection

We collected two milliliters of blood from the antecubital fossa and then dispensed it into a sterile tube containing ethylene diamine tetraacetic acid. To prevent clot formation, we thoroughly mixed the blood. DNA isolation following the modified protocol was established by Miller et al. in 1988 [16].

Polymerase chain reaction and restriction endonuclease digestion

**Figure 1:**
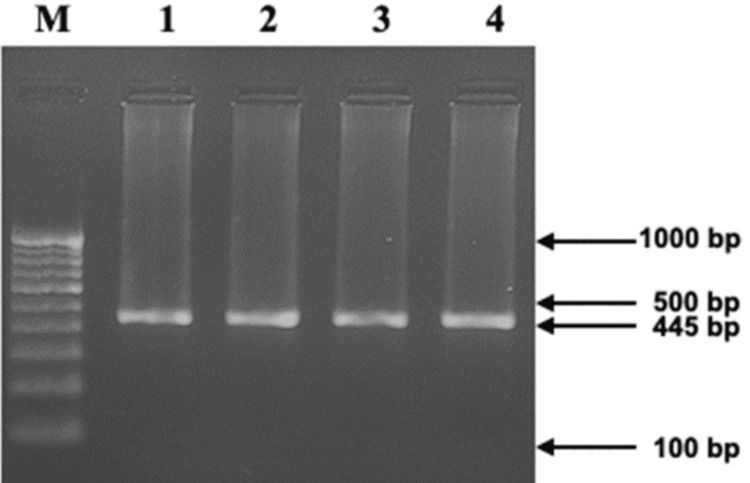
Agarose gel electrophoretogram displaying the MMP-13 (rs2252070) gene polymorphism, revealing a 445 bp amplicon in lanes 1-4 (Lane [M]: 100 bp DNA ladder).

**Figure 2:**
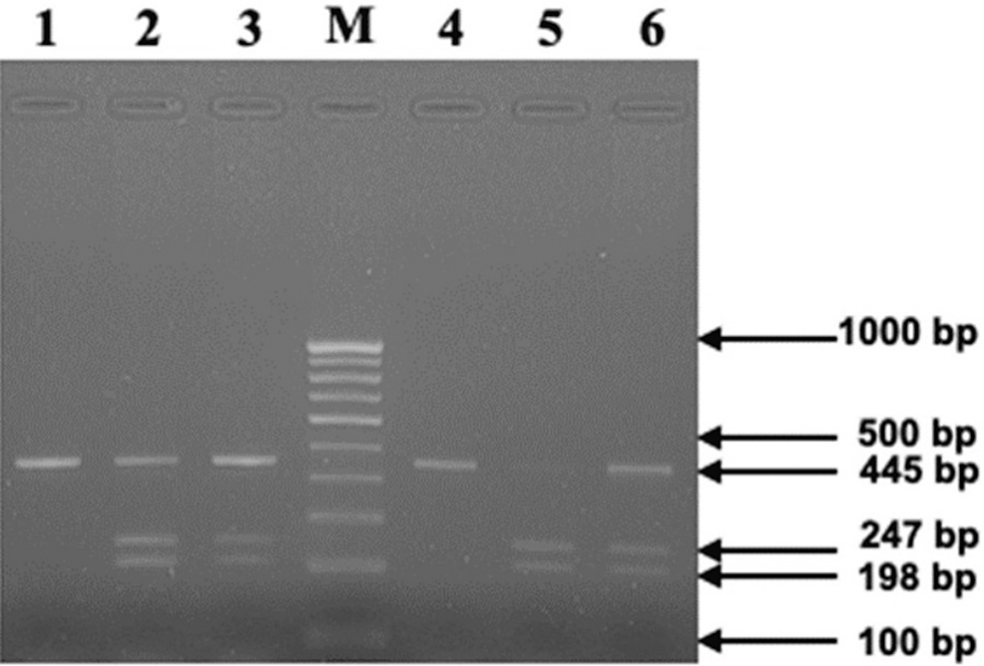
BsrI digestion (Lanes 1-GG homozygous variant, 2, 3, 6 AG heterozygous, 4-AA-homozygous wild type).

We evaluated MMP-13 gene polymorphism (rs2252070) through PCR amplification followed by subsequent digestion. 5’-GATACGTTCTTACAGAAGGC-3’ was the forward primer, and the reverse primer was 3’-ACAAATCATCTTCATCACC-5’, which spanned the polymorphic site of the MMP-13 gene and was used for DNA amplification. We employed a polymerase chain reaction (PCR) master mix from Takara, Japan, to amplify DNA in 20 μL volumes. The amplification reaction comprised 10 ng of genomic DNA; the quantity of the forward and reverse primers was 5 pmol/μL each, along with an additional 10 ng of genomic DNA. Initially, denaturation was conducted for five minutes at 94 °C, then denaturation at 94 °C for 35 seconds. For another 35 seconds, annealing was conducted at 60 °C, extension at 72 °C for 35 seconds, and a final extension for five minutes at 72 °C. Following this process, we used a 2% agarose gel to analyze 5 μL of the PCR product. We used the BsrI restriction enzyme (New England Biolabs, England) for carrying out the digestion of a 15-microliter volume of the PCR product at 37 °C for two hours. The results of the visualization of the digested material on a 2.5% agarose gel are shown in Figure 1 and Figure 2.

Statistical analysis

We used IBM SPSS Statistics for Windows, version 23.0 (released 2015, IBM Corp., Armonk, NY) in the current study. We conducted the Chi-square test for comparison of genotype and allele frequency distributions between the periodontitis and control groups. We calculated 95% confidence intervals and the odds ratio to evaluate the risk associated with specific genotypes or alleles. P < 0.05 was utilized in each test to determine statistical significance.

## Results

A total of 100 patients were included in the study: 50 in the periodontitis group and 50 in the healthy group. Each group comprised of 26 females and 24 male patients. The mean age of the participants was 36.44 ± 4.32 years for the 50 patients in the periodontally affected group. In the healthy controls, the mean age was 38.02 ± 4.26 for the 50 individuals exhibiting either mild gingivitis or a healthy periodontium. All the patients were diagnosed based on the AAP 2018 criteria by two separate examiners. The mean clinical attachment loss was recorded as 5.23 ± 1.44 in the periodontitis group. The recorded probing pocket depth was in the range of 5.56 ± 0.35 in the periodontitis group and 1.34 ± 0.27 in the healthy controls. The mean gingival index was recorded as 1.34 ± 0.38 in the periodontitis group, and it was 0.33 ± 0.04 in the controls.

The genotype frequency and distribution of MMP-13 polymorphism did not exhibit a statistically significant difference at χ2 degrees of freedom (P = 0.913) for the AA, AG, and GG genotypes and for the A and G alleles. The current study indicated no significant difference between the two groups concerning homozygous and heterozygous mutant genotypes (AA vs. AG + GG), with a P-value of 0.6871. The observed frequencies of GG (47% vs. 43%) and AG+AA (41% vs. 42%) genotypes did not indicate any significant difference between the groups. Similarly, there was no noteworthy distinction in the A allele (62% vs. 65%) or G allele (38% vs. 35%) between the diseased and control groups. Non-significant results were obtained when checked for all alleles. The MMP-13 genotype distribution for the dominant and recessive alleles thus indicated that there was no noteworthy correlation between the gene polymorphism and the presence of periodontitis. Table 1 and Table 2 present genotype and allele frequency data for the respective case and control groups.

**Table 1:** Genotype frequencies of the MMP-13 (rs2252070) gene polymorphism in the case group and control group

Groups	AA	AG	GG	A	G	HWE (p-value)*
Case (N = 50)	21	20	9	0.62	0.38	0.2853
Control (N = 50)	23	19	8	0.65	0.35	0.2437

**Table 2:** MMP-13 (rs2252070) genotype distribution in the case group and control group. OR: odds ratio; CI: confidence interval

Dominant				
Genotypes	Case	Control	Unadjusted OR [95% CI]	P value
AA	21	23	0.8501 [0.3857–1.8738]	0.6871
AG + GG	29	27		
Recessive				
GG	9	8	1.1524 [0.4053–3.2771]	0.7902
AG + AA	41	42		
Allele				
A	62	65	0.8785 [0.4938 - 1.5630]	0.6595
G	38	35		

## Discussion

In periodontitis, the degradation of connective tissues and the alveolar bone occurs, which is primarily instigated and sustained by a majority of gram-negative anaerobic groups of bacteria. Although it is widely recognized that bacteria initiate the disease, their presence alone is insufficient to determine how the disease will progress and what outcomes will be reached. The severity of the disease is influenced by interactions between the host and bacteria, which can be altered by various risk factors, including environmental, acquired, and genetic factors.

Recent research indicates that genetic factors contribute to nearly 50% of the variations in the clinical characteristics of chronic periodontitis [17,18]. It is well established that genetic variations can impact the quality and quantity of the host's response to microbial challenges. Exploring the connection between these genetic polymorphisms and the consistent phenotypic characteristics of patients with periodontitis could provide the foundation for identifying molecular biomarkers that can be integrated into individual risk assessments. This research may also contribute to developing innovative treatment approaches [19]. A substantial body of evidence supporting a genetic component in the development of periodontitis derives from numerous studies conducted on aggressive periodontitis [19]. Notably, research focused on this specific type of periodontitis is likely to reveal the genetic variations associated with the condition, providing valuable insights that can enhance the management of patients in clinical settings.

MMP-13 plays a vital role in the breakdown of collagen fibers. Osteoblasts are the primary cellular source of its expression [20]. Upregulation of the MMP-13 enzyme is primarily conducted by the parathyroid hormone, which resorbs bone. In cases of normal tissues, either minimal or no activity has been seen in terms of MMP-13 [21]. Because MMP-13 is capable of causing the degradation of both type I and II collagen, it can be said that it has an important role in bone resorption, making it a notable target in inflammatory bone diseases [22]. Because it has a degradative activity and exhibits a greater expression in inflammatory bone diseases, it is imperative to understand its expression and regulation.

This study has assessed the potential involvement of MMP-13 gene polymorphisms in individuals with periodontitis compared to healthy individuals. The outcomes of this study indicate that there was no statistically significant difference in the genotype frequency and distribution of MMP-13 polymorphism at χ2 degrees of freedom (P = 0.913). We found no significant difference between the case and control groups concerning homozygous and heterozygous mutant genotypes (AA vs. AG + GG), with a P-value of 0.6871. The genotype frequencies of GG (47% vs. 43%) and AG+AA (41% vs. 42%) also showed no significant difference between the groups. Similarly, we noted no significant difference in the A allele (62% vs. 65%) or G allele (38% vs. 35%) between the two groups. Previously, a study assessing the GCF MMP-13 level correlation with periodontal therapy reported no significant difference associated with 11A/12A in both periodontally compromised individuals and those free of disease, which is consistent with the findings of our study [23]. Hernandez et al. saw a 100% link between MMP-13 in gingival crevicular fluid samples from patients diagnosed with chronic periodontitis (CP) and found that higher quantities of MMP-13 were associated with active sites compared with inactive sites [13]. Hernández-Ros et al. discovered that during disease progression in the case of CP, MMP-13 could degrade both soft and hard tissues [24].

Many researchers have analyzed the link between other MMP polymorphisms and the risk of periodontitis, but the final results remain inconclusive or unclear. MMP-13 belongs to the collagenases group, specifically referred to as collagenase-3 [25]. The initial step of the breakdown of interstitial collagens by collagenases, such as MMPs-1, 8, and 13, is recognized as vital to the healing process of periodontal lesions. MMP activity contributes to the pace of matrix turnover and immunologic response modulation through more direct actions [26]. MMP-13 is present in small amounts or is virtually absent in gingival tissues free of disease, indicating a very limited distribution pattern [14]. The advancement of disease activity might be linked to the imbalance between MMP-13 and its inhibitors, resulting in elevated enzyme activity. The indication of MMP-13 expression in alveolar bone loss has been proposed as a characteristic of periodontitis progression [27].

In our current investigation, we have presented information on the genetic polymorphism at -1607 bp in MMP-13 among a cohort of South Indian individuals. Our results indicate that the MMP-13 polymorphism is not linked to periodontitis in the examined population.

Limitations

It is crucial to initiate more extensive studies with a predominant sample size, improved techniques, and the inclusion of diverse ethnic populations. Conducting such studies could provide insights into the potential relevance of MMP-13 gene polymorphisms and their correlation to periodontitis.

## Conclusions

Despite numerous extensive studies examining the link between gene polymorphisms and the vulnerability and/or severity of periodontitis, a considerable level of inconsistency persists, and the outcomes remain inconclusive. Although there has been significant progress in comprehending genetic risk factors for periodontal disease, the genetic basis is still being elucidated. The investigation of allelic variants of genes utilized to assess the risk of periodontal disease has garnered interest. However, as of now, only a limited number of genetic polymorphisms have been reported. Our study suggests that there is no association between MMP-13 (rs2252070) gene polymorphism and periodontitis (classified as stage II/III, grade B) in the analyzed population. However, additional investigations are necessary to delve into a possible association between this gene polymorphism and the etiopathogenesis of the disease.
